# Rinderpest Virus Sequestration and Use in Posteradication Era

**DOI:** 10.3201/eid1901.120967

**Published:** 2013-01

**Authors:** Guillaume Fournié, Wendy Beauvais, Bryony A. Jones, Juan Lubroth, Francesca Ambrosini, Félix Njeumi, Angus Cameron, Dirk U. Pfeiffer

**Affiliations:** Author affiliations: Royal Veterinary College, London, UK (G. Fournié, W. Beauvais, B.A. Jones, D.U. Pfeiffer);; Food and Agriculture Organization of the United Nations, Rome, Italy (J. Lubroth, F. Ambrosini, F. Njeumi);; AusVet Animal Health Services, Wentworth Falls, New South Wales, Australia (A. Cameron)

**Keywords:** Rinderpest, eradication, virus sequestration, viruses, ruminants, vaccine

## Abstract

After the 2011 declaration of rinderpest disease eradication, we surveyed 150 countries about rinderpest virus stocks. Forty-four laboratories in 35 countries held laboratory-attenuated strains, field strains, or diagnostic samples. Vaccine and reagent production and laboratory experiments continued. Rigorous standards are necessary to ensure that stocks are kept under safe conditions.

During 2011, a major milestone in global infectious disease control was achieved. Rinderpest was declared eradicated by the Food and Agriculture Organization of the United Nations (FAO) and the World Organisation for Animal Health (OIE). Rinderpest is a disease caused by rinderpest virus, a paramyxovirus of the genus *Morbillivirus*; the disease has had a devastating effect on livestock health, productivity, and welfare. It became the second disease to be eradicated, 30 years after smallpox eradication. The last reported outbreak of rinderpest occurred during 2001 in Kenya (*1*).

Unintentional or deliberate virus release remains a serious concern as long as virus-containing material remains in the possession of vaccine manufacturers or research and diagnostic laboratories. An example to substantiate this concern is the last reported case of smallpox in a human, which originated from a laboratory (*2*). To manage the risk for rinderpest reintroduction, identifying these potential virus sources is essential. We conducted a questionnaire survey to assess the location and number of rinderpest virus stocks, their uses, and their storage conditions.

## The Study

We developed 2 password-protected online questionnaires, 1 for national veterinary authorities and 1 for laboratory staff. Questionnaires were available in the 6 official languages of the United Nations: English, French, Spanish, Russian, Arabic, and Chinese. A snowball sampling approach was adopted. Questionnaires were first sent in May 2011 to 192 national veterinary authorities and to 126 laboratories for which contact details were known. National veterinary authorities were asked to forward the laboratory questionnaire to all government, university, and private laboratories that might be involved in cattle virus diagnostics, research, or vaccines within their countries. Laboratory personnel were also asked to forward the survey to other laboratories. Questionnaire responses were inconsistent for 4 survey participants, who were then contacted for clarification. Survey data were treated confidentially.

This survey captured data from 100 countries (81 national veterinary authorities and 100 laboratories). It was complemented with the results of a previous questionnaire survey undertaken during 2010 by the FAO and OIE to identify countries holding rinderpest virus stocks and with information obtained from unpublished, or “grey,” literature and direct discussions with laboratory staff. As a result, information was available for 150 (76%) of the 198 OIE or FAO member and associate member countries (Figure).

**Figure:**
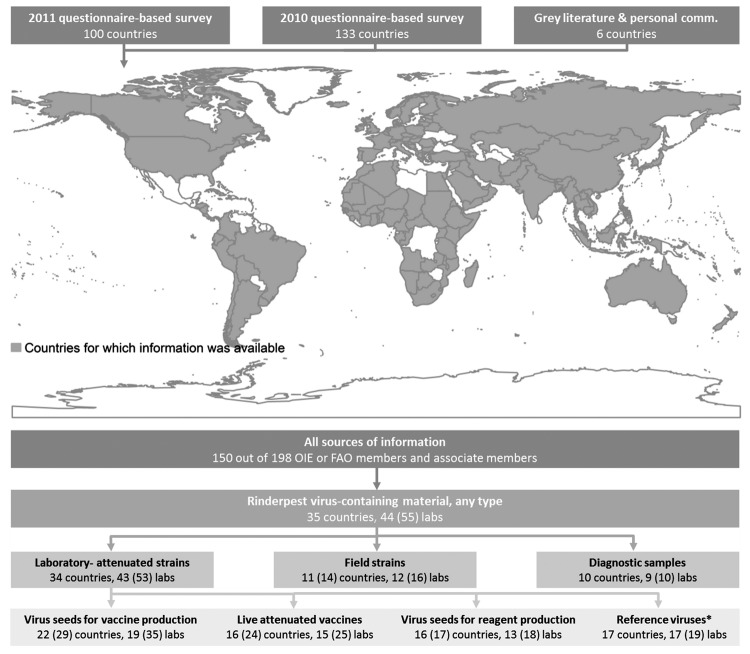
Countries and laboratories holding material containing rinderpest virus, June 2011. Numbers in parentheses indicate the potential number of laboratories or countries holding a given type of virus when accounting for incomplete information. *Reference virus or specimens as part of a collection for research or teaching. FAO, Food and Agriculture Organization of the United Nations; OIE, World Organisation for Animal Health.

Thirty-five countries held rinderpest virus–containing material: 37% in Asia, 29% in Africa, and 26% in Europe. Extrapolating these results for countries for which information was not available, we estimated a global tally of 41 (95% credible interval 37–47) countries that held rinderpest virus (Technical Appendix).

A susceptible host infected by a laboratory-attenuated strain cannot become infectious unless mutations facilitating virus reversion to transmissibility occur during infection. Laboratory-attenuated strains were the most commonly held virus type, kept in 34 countries, mainly as live vaccines or virus seed for vaccine production. Live vaccine stocks could be quantified for 11 countries, 4 of which had 100,000–4,000,000 doses. Field strains, which included mild to virulent strains that are transmissible between hosts, were held in 11 countries. We also received incomplete information; for example, several laboratory personnel reported holding virus in the past but did not specify whether their laboratories still held it. When we accounted for this incomplete information, we determined that 14 countries could be holding field strains (Figure). Diagnostic samples, which included blood and tissues from animals infected or suspected to be infected, were held in 10 countries.

Rinderpest virus manipulations were being performed by some countries. A total of 115,000 live vaccine doses were produced in 1 country in 2011, and diagnostic reagent tests have been produced by using live virus in 3 countries since 2010. Basic research was still being conducted in 3 countries, of which 1 was conducting in vivo experiments on rabbits using laboratory-attenuated strains. Laboratories in some countries stated that they planned to develop marker and recombinant vaccines and to maintain and upgrade diagnostic reagent stocks. The sequencing of field strains was also being considered.

We identified 44 laboratories holding viruses. Among the 36 laboratories for which biosafety level (BSL) was known, 11 (31%) of 36 laboratories holding attenuated strains were classified as BSL-2 or lower, as were 2 (17%) of 12 laboratories holding field strains and 3 (38%) of 8 laboratories holding diagnostic samples. Nine (39%) of 23 laboratories reported providing veterinary authorities with an inventory of the material containing rinderpest virus that were held, and 3 (13%) laboratories reported that authorization from veterinary authorities was required before they could manipulate rinderpest viruses.

Fourteen national veterinary authorities reported holding a complete and up-to-date inventory of all rinderpest virus for all laboratories within the country. However, 43% of them did not report all of the virus types that were reported as being held by the laboratories themselves.

Veterinary authorities of the 66 countries where rinderpest vaccines were used during the past 30 years were invited to forward a separate online questionnaire to field veterinarians, to assess their awareness of rinderpest and vaccine use. Of 70 respondents from 21 countries, none reported any use of rinderpest vaccines after 2003; however, 36% of respondents from 9 countries estimated that the likelihood of using rinderpest vaccines for routine vaccination against rinderpest or peste des petits ruminants, another morbillivirus disease, in their region within the next 5 years was “possible” to “very likely.”

## Conclusions

Because data were not available for all countries or all laboratories within countries, the number of virus stocks was probably underestimated. Some laboratory staff could retain material containing rinderpest virus without having ever conducted research on rinderpest or reported their stocks to their national authority. These laboratories might have been missed by our sampling approach. The number of respondents might have been greater if we had made questionnaires available in other languages. However, despite the sampling limitations, we can conclude that a large number of countries hold material containing rinderpest virus.

Some veterinary authorities did not report all the virus types held in their countries, despite most of them claiming to maintain an inventory. Unless all virus stocks in all countries can be assessed, virus sources could be missed by future relocation plans.

The reasons given for conducting basic research on rinderpest included that rinderpest viruses were a good model for studying aspects of morbilliviruses, such as mechanisms of replication, host pathogenicity, and interactions between host cell factors and viruses. Although such research might still be relevant and the production and management of vaccine and diagnostic reagent stocks could be key components of contingency plans, modifications to laboratory activities and protocols are needed to meet compliance standards recently recommended by FAO and OIE (*3*). Under these new standards, all laboratories holding any virus stocks must meet BSL-3 criteria, and manipulations involving rinderpest virus must be approved by the national veterinary authority, as well as by FAO and OIE. Adhering to the new standards and subjecting activities to approval also applies to the potential reconstruction of rinderpest virus de novo, given that the technical capacity and genome sequences are available.

Some field veterinarians reported believing that rinderpest vaccines might be used in their region in the near future. We did not investigate the reasons behind these opinions, but awareness clearly needs to be raised in the veterinary sector about risks for rinderpest reintroduction despite eradication and, more specifically, about the restricted use of rinderpest vaccines solely for the management of confirmed rinderpest outbreaks (3).

Technical Appendix Estimation of the total number of countries holding rinderpest viruses.
