# Supplementary material for: Rinderpest Virus Sequestration and Use in Posteradication Era
Source: Emerg Infect Dis. 2013 Jan;19(1):151–3. doi: 10.3201/eid1901.120967 (PMC3557999; doi:10.3201/eid1901.120967)
Supplement: Technical Appendix — Estimation of the total number of countries holding rinderpest viruses. [file 12-0967-Techapp-s1.pdf]

Article DOI: http://doi.dx.org/10.3201/eid1901.120967

Rinderpest Virus Sequestration and Use in Posteradication Era

Technical Appendix

Estimation of the total number of countries holding rinderpest viruses

When assessing the total number of countries holding rinderpest viruses, simulations were performed to estimate the status of countries for which no information was available. Based on characteristics presumed to influence a country's likelihood of holding rinderpest viruses, all countries were classified into 1 of 4 groups (see groups described below) or 1 of 3 groups (where the last 2 groups described below were merged into 1):

- Countries (n = 22) in which rinderpest vaccines have been produced, or research activities have been recently conducted (i.e., countries from which scientific papers involving in vivo or in vitro experiments with rinderpest viruses have been published over the past 10 years and are listed on Pubmed, www.ncbi.nlm.nih.gov/pubmed/).
- Countries (n = 39) in which vaccine production and research activities have not been recently conducted, but which have a recent rinderpest history (rinderpest outbreak since 1983 and/or vaccination since 1998).
- Countries (n = 17) in which vaccine production and research activities have not been recently conducted, which have not experienced outbreaks since 1983 and have not vaccinated since 1998, but which experienced outbreaks between 1950 and 1983.
- All other countries (n = 120).

Each group, i, was formed of N_i countries. Among them, n_i countries were surveyed, of which y_i were found to hold rinderpest virus–containing material. The surveyed countries were assumed to be a random sample from each group. Moreover, it was assumed that the surveyed countries were accurately classified as virus holders or virus nonholders. The number of countries in possession of rinderpest virus–containing material, Y_i, within a group i was estimated using Bayesian inference, as follows:

$$f(Y_i \mid n_i, y_i, N_i) \propto \pi(Y_i)\, l(y_i \mid n_i, Y_i, N_i)$$

with $f(Y_i \mid n_i, y_i, N_i)$ being the posterior distribution, equal here to the probability distribution of Y_i. This distribution depended on the prior distribution of Y_i, $\pi(Y_i)$, which was assumed to be uniform, and the likelihood function $l(y_i \mid n_i, Y_i, N_i)$. The latter was a hypergeometric probability mass function with parameters being the number of successes, y_i, from n_i draws from a population of size N_i, which itself included Y_i successes.

The probability distribution of the total number of countries holding rinderpest virus–containing material in the world, Y, was assessed through simulations. At each iteration, a value of Y_i was randomly sampled for each group i from their respective probability distributions. These values were then summed, giving a simulated estimate of Y. Such iterations were repeated 100,000× to produce the probability distribution of Y. The mean estimate and the 95% credible interval (CrI) are shown.

Based only on the 2011 survey results, the total number of countries having virus stocks was estimated to be 41 (95% CrI 33–52). When using all available information, the total number of countries holding viruses was also estimated to be 41 (95% CrI 37–47) (Technical Appendix Table).

Technical Appendix Table. Estimation of the number of countries holding rinderpest viruses

Data source	Countries partitioned into 3 groups (95% credible interval)	Countries partitioned into 4 groups (95% credible interval)
National veterinary authority questionnaires	39 (30–50)	40 (31–51)
National veterinary authority and laboratory questionnaires	41 (33–51)	41 (33–52)
All sources of information	41 (37–47)	41 (37–47)
